# Rhamnan sulphate from green algae *Monostroma nitidum* improves constipation with gut microbiome alteration in double-blind placebo-controlled trial

**DOI:** 10.1038/s41598-021-92459-7

**Published:** 2021-07-05

**Authors:** Yasuhito Shimada, Masahiro Terasawa, Fumiyoshi Okazaki, Hiroko Nakayama, Liqing Zang, Kaoru Nishiura, Koichi Matsuda, Norihiro Nishimura

**Affiliations:** 1 Department of Integrative Pharmacology, Mie University Graduate School of Medicine, 2-174 Edobashi, Tsu, Mie 514-8507 Japan; 2 Mie University Zebrafish Drug Screening Center, Tsu, Mie 514-8507 Japan; 3 Department of Bioinformatics, Mie University Advanced Science Research Promotion Center, Tsu, Mie 514-8507 Japan; 4 Graduate School of Bioresources, Mie University, Tsu, Mie 514-8507 Japan; 5 Graduate School of Regional Innovation Studies, Mie University, Tsu, Mie 514-8507 Japan; 6 Konan Chemical Manufacturing Co., Ltd., Yokkaichi, Mie 510-0103 Japan

**Keywords:** Applied microbiology, Microbial communities, Microbiota, Intestinal diseases, Drug discovery

## Abstract

Rhamnan sulphate (RS), a sulphated polysaccharide from *Monostroma nitidum*, possesses several biological properties that help in treating diseases such as viral infection, thrombosis, and obesity. In the present study, we first administered RS (0.25 mg/g food volume) orally to high-fat diet-treated mice for 4 weeks. RS increased the faecal volume and calorie excretion with decreased plasma lipids, which was in accordance with the results of our previous zebrafish study. Notably, as the excretion amount by RS increased in the mice, we hypothesised that RS could decrease the chance of constipation in mice and also in human subjects because RS is considered as a dietary fibre. We administrated RS (100 mg/day) to subjects with low defaecation frequencies (3–5 times/week) for 2 weeks in double-blind placebo-controlled manner. As a result, RS administration significantly increased the frequency of dejection without any side effects, although no effect was observed on the body weight and blood lipids. Moreover, we performed 16s rRNA-seq analysis of the gut microbiota in these subjects. Metagenomics profiling using PICRUSt revealed functional alternation of the KEGG pathways, which could be involved in the therapeutic effect of RS for constipation.

## Introduction

Seaweeds contain high levels of iodine, iron, vitamin C (which aids iron absorption), anti-oxidants, soluble and insoluble fibre, vitamin K, vitamin B-12, and are well-known natural reserves of polysaccharides^1^. Sulphated polysaccharides are most common in the cell wall of seaweeds, and their dietary consumption is expected to impart potential benefits for human health, including improvement of obesity and gut dysbiosis. Of these, fucoidan and carrageenan are beneficial sulphated polysaccharides, which exhibit anti-obesity properties through altering the composition of gut microbiota^2^. These polysaccharides were extensively studied using high fat diet-induced obese mice and clinical trials with obese population, and these studies proved that their anti-obesity properties were involved in the reduction of *Bacteroidetes*/*Firmicutes* ratio^3^, which is a relevant marker of gut dysbiosis in obese population, increased diversity of microbiota^4,5^, and increase in specific gut bacteria^5,6^.

Green algae belong to *Monostroma* genus, and are commercially cultivated in East Asia and South America for edible purposes, as popular sushi wraps. Rhamnan sulphate (RS) is a sulphate polysaccharide comprising l-rhamnose and sulphated l-rhamnose found in green algae, and was purified as the main constituent from the cell walls of *Monostroma lattisimum and Monostrom nitudum* in 1998^7^, as an activator for anti-thrombin^8^. For the following 20 years, several biological activities of RS have been identified such as its anti-coagulant^9–12^ and anti-viral effects^13–16^ and others^17^. Of these, we discovered lipid-lowering properties of RS to improve hepatic steatosis, using a diet-induced obesity model of zebrafish in 2015^18^.

Similar to other multifunctional polysaccharides^19,20^, RS is also expected to exhibit a variety of therapeutic functions, but its anti-obesity function has not been evaluated in mammals and humans yet. In the present study, to validate the anti-obesity properties of RS in mammals, we administered RS to high-fat diet-induced obese mice and found that RS increased the excretion amount and calories with lipid-lowering effects. Furthermore, we performed a clinical trial on subjects with constipation tendency to determine whether RS can be used as a therapeutic molecule.

## Results

### Rhamnan sulphate (RS) increases faecal amount in obese mice

**Figure 1:**
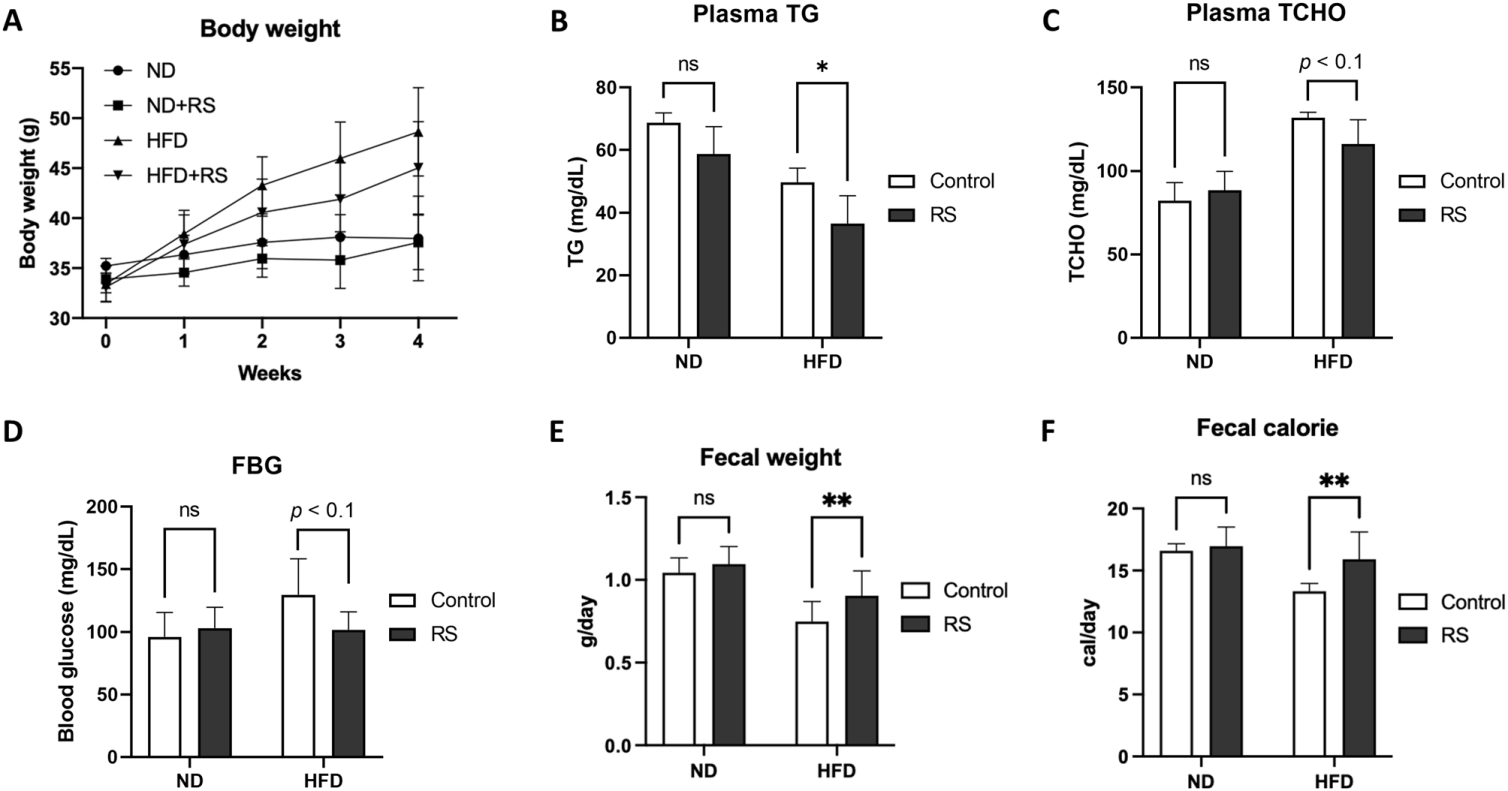
RS decreases blood lipids and increases faecal amount in mice with high fat diet (HFD). (**A**) Body weight change during the feeding experiment. (**B,C**) RS suppression increases in plasma triacylglycerol (TG; (**B**)) and total cholesterol (TCHO; (**C**)) in HFD-mice at Week 4. (**D**) Fasting blood glucose (FBG) at Week 4. (**E,F**) RS increased in faecal weight (**E**) and faecal calorie (**F**) in HFD-mice during the feeding experiment. *p < 0.05, **p < 0.01. *n* = 5, error bars indicate SD.

After 4-week feeding, RS revealed a tendency (*p* < 0.1) to suppress body weight increase in the HFD group (48.6 ± 4.4 g in HFD vs. 45.0 ± 4.6 g in HFD + RS group; Fig. 1A). Corresponding to the suppression of body weight, plasma triglycerides (49.8 ± 4.4 mg/dL in HFD vs. 36.6 ± 8.9 mg/dL in HFD + RS group; Fig. 1B) and total cholesterol (131.9 ± 3.2 mg/dL in HFD vs. 116.1 ± 14.6 mg/dL in HFD + RS group; Fig. 1C) were significantly (*p* < 0.05) suppressed by RS. Moreover, RS significantly (*p* < 0.05) suppressed fasting blood glucose (129.8 ± 28.7 mg/dL in HFD vs. 101.8 ± 14.4 mg/dL in the HFD + RS group; Fig. 1D). For faecal analysis, RS significantly (*p* < 0.01) increased the faecal weight (0.75 ± 0.12 g/day in HFD vs. 0.90 ± 0.15 g/day in HFD + RS group; Fig. 1E) and calories (13.3 ± 0.6 kcal/day in HFD vs. 15.9 ± 2.2 kcal/day in HFD + RS group; Fig. 1F) in HFD groups.

### RS improved constipation in subjects with low defaecation frequency

**Figure 2:**
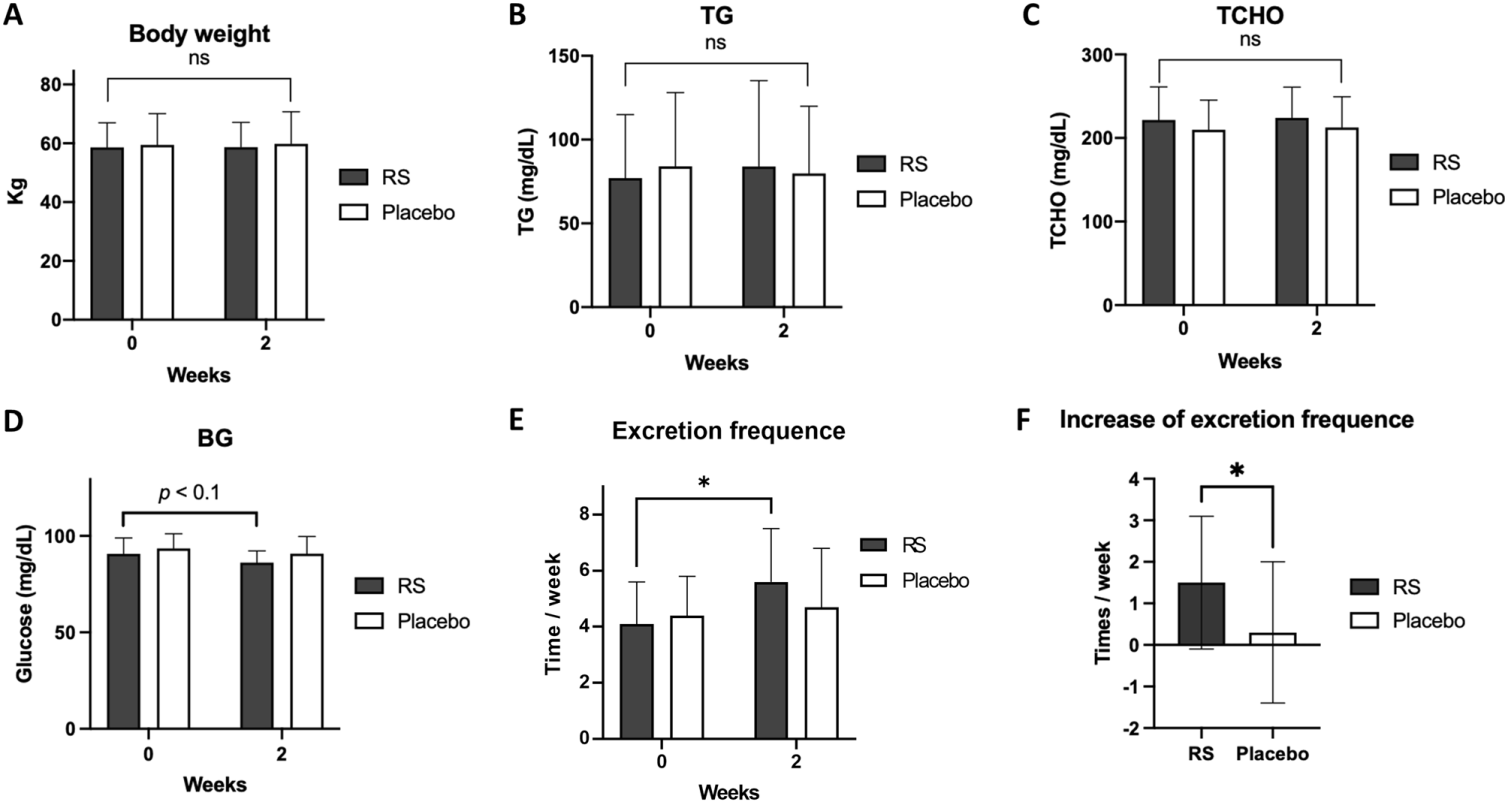
RS increases defaecation in human subjects. (**A**) Body weight change during the trial. (**B–D**) Plasma triacylglycerol (TG; (**B**)), total cholesterol (TCHO; (**C**)) and blood glucose (BG; (**D**)) at Weeks 0 and 4. (**E,F**) RS increased in faecal frequency at Week 2. *p < 0.05, **p < 0.01. *n* = 19, error bars indicate SD.

From the mouse experiment, we hypothesised that RS has therapeutic properties to improve constipation, thereby subsequently decreasing blood lipids and body weight. Thus, we performed a clinical trial with chronic constipation (Fig. S1). The present study was performed from 28th February to 23rd April 2020. Seventy-three volunteers were initially screened, as illustrated in Fig. S2. The final 38 healthy volunteers (participants), who had relatively low defaecation frequencies (3–5 times a week), were randomly allocated to the groups to receive RS or placebo. The baseline characteristics of the participants are summarized in Table 1. No significant difference was observed between RS and the placebo group for any baseline characteristics (*p* ≥ 0.05). In contrast to the mouse experiment, RS did not decrease the body weight (Fig. 2A), plasma TG (Fig. 2B), TCHO (Fig. 2C); Blood glucose exhibited a decreasing trend (*p* < 0.1) upon RS administration (90.7 ± 8.3 mg/dL at 0 week vs. 86.2 ± 6.0 mg/dL at 2 weeks in the RS group; Fig. 2D), which was in accordance with that of the results with mouse experiment (Fig. 1D). RS significantly (*p* < 0.05) increased the excretion frequency (4.1 ± 1.5 time/week at 0 week vs. 5.6 ± 1.9 time/week at 2 weeks in RS group; Fig. 2E) with increase in the excretion frequency from 0 to 2 weeks **(**0.3 ± 1.7 time/week in placebo group vs. 1.5 ± 1.6 time/week in RS group at 2 weeks; Fig. 2F). Moreover, the excretion days per week were also significantly (*p* < 0.05) increased by RS administration (3.8 ± 1.0 days/week at 0 week vs. 4.9 ± 1.2 days/week at 2 weeks in the RS group) with increase in the excretion days from 0 to 2 weeks (0.3 ± 1.5 days/week in placebo group vs. 1.1 ± 1.3 days/week in RS group at 2 weeks; Fig. S3). Presumably, these results are similar to those observed in our mouse study (Fig. 1E,F). No important harms or unintended effects was observed during the study.

**Table 1:** Base line characteristics in human subjects.

Items	RS group	Control group	*p* value
Age (years)	48.6 ± 8.4	49.1 ± 7.2	0.8691
Sex (male/female)	8/11	9/10	1.0000
Height (cm)	166.51 ± 9.55	163.98 ± 5.96	0.3345
Body weight (kg)	58.89 ± 8.35	59.97 ± 10.31	0.7238
BMI (kg/m^2^)	21.18 ± 1.84	22.23 ± 3.18	0.2207
Systolic blood pressure (mmHg)	114.9 ± 10.7	118.1 ± 10.1	0.3559
Diastolic blood pressure (mmHg)	70.8 ± 8.8	75.8 ± 7.9	0.0760
Pulse rate (beats/min)	66.7 ± 7.2	70.3 ± 8.2	0.1560
Excretion frequency (times/week)	3.9 ± 0.8	3.9 ± 0.8	1.0000

RS (n = 19), Control (n = 19).

Mean ± SD.

*p*-value: unpaired t-test.

### RS alters microbiota composition in subjects

**Figure 3:**
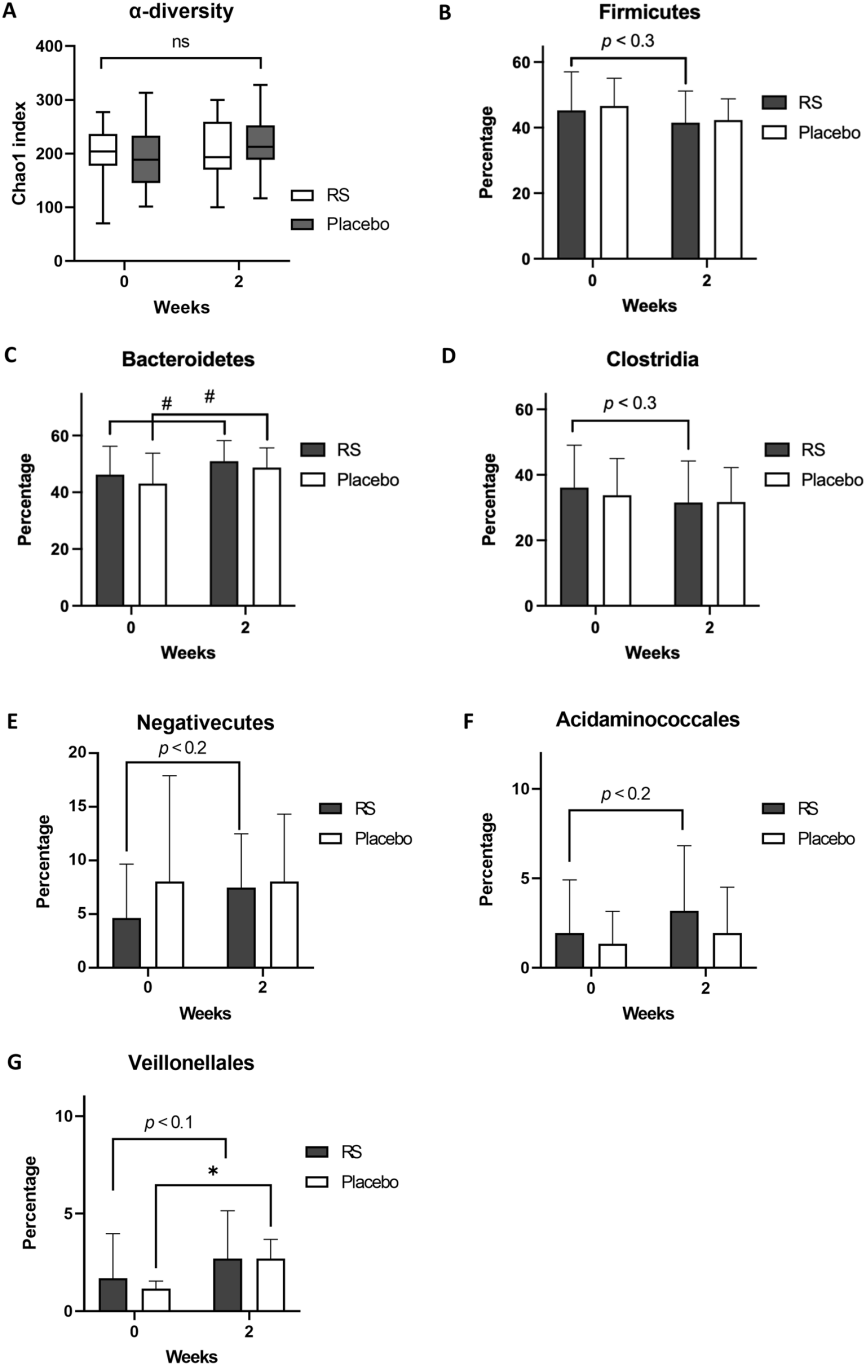
Difference in bacterial composition between RS and placebo group. (**A**) α-diversity analysis. (**B**,**C**) *Firmicutes* (**B**) and *Bacteroidetes* (**C**) alteration during the trial. (**D,E**) In *Firmicutes* phylum, *Clostridia* class showed a tendency (*p* < 0.3) to decrease in RS group (**D**), whereas *Negativicutes* class increased (*p* < 0.2) in RS (**E**). (**F,G**) In *Negativicutes* class, *Acidaminococcales* order increased in RS group (**F**), whereas *Veilonellales* increased in both groups (**G**). ^#^*p* < 0.1, *p < 0.05. *n* = 19, error bars indicate SD.

The taxonomy summary of the clinical trials is described in Figs. S4–S6. We also evaluated the α-diversity using the Chao1 index (Fig. 3A; no difference between groups) and β-diversity using the UniFrac matrix (Supplementary Movie). At the phylum level, Firmicutes revealed a decreasing tendency due to RS (*p* < 0.3; Fig. 3B), whereas Bacteroidetes increased in both RS (*p* < 0.1: 46.2% ± 10.0% at 0 week vs. 51.0% ± 7.2% at 2 weeks) and placebo groups (43.1% ± 10.7% in 0 week vs. 48.7% ± 6.9% at 2 weeks; Fig. 3C). For *Clostridia*, a major class in the phylum *Firmicutes*, RS exhibited a tendency (*p* < 0.3) to decrease their proportion (Fig. 3D). RS exhibited a tendency (*p* < 0.2) to increase *Negativicutes* (4.6% ± 5.0% at 0 week vs. 7.5% ± 5.0% at 2 weeks in the RS group; Fig. 3E). In *the Negativicutes* class, we detected four orders, and two of these, *Acidaminococcales* (1.9% ± 3.0% at 0 week vs. 3.2% ± 3.6% at 2 weeks in the RS group; Fig. 3F) and *Veillonellales* (1.7% ± 2.3% at 0 week vs. 2.7% ± 2.5% at 2 weeks in the RS group; Fig. 3G), were slightly increased by RS.

### Sub-cluster analysis reveals RS sensitive subjects

**Figure 4:**
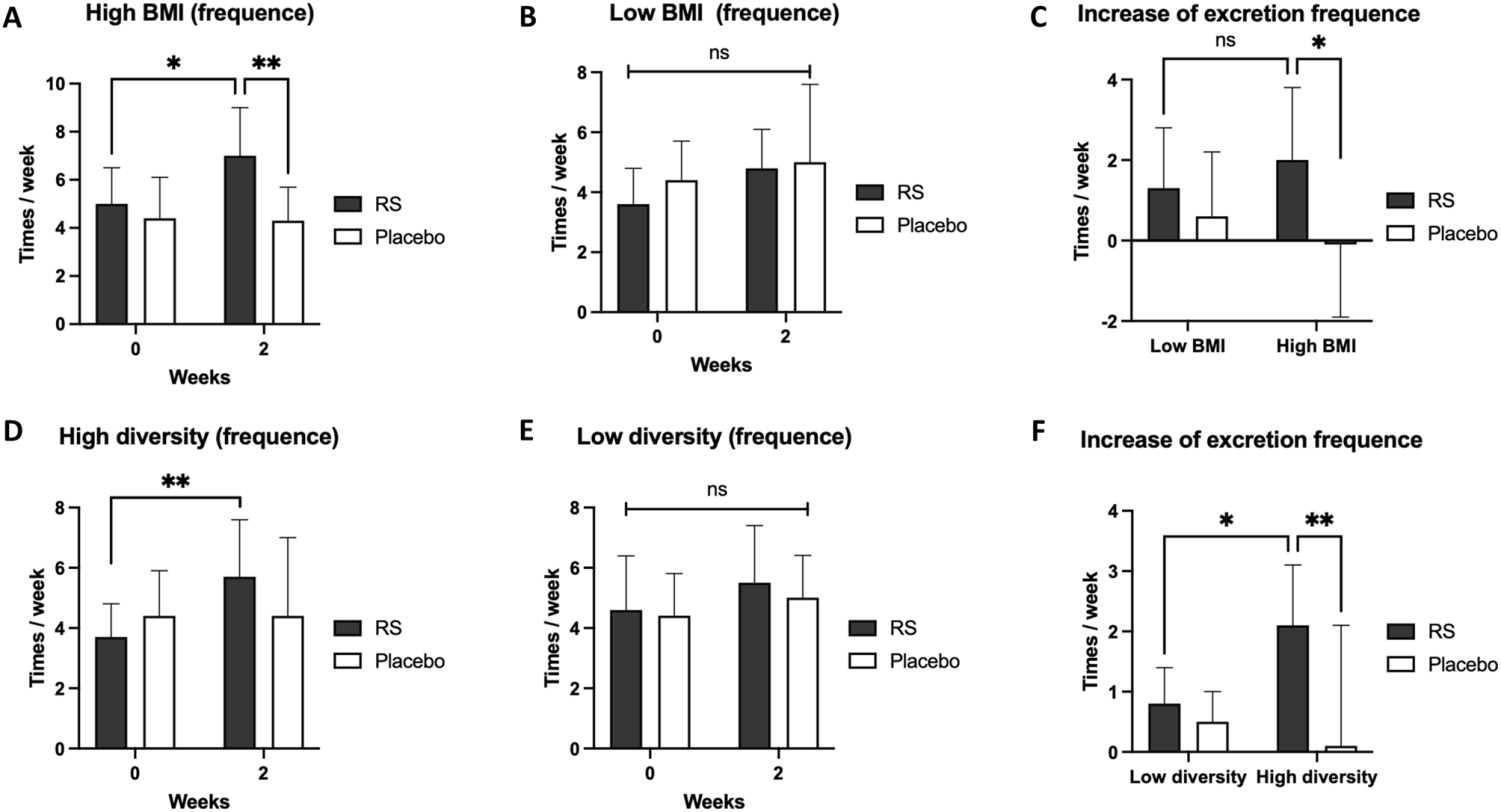
Sub-cluster analysis in human subjects. (**A**,**B**) In high BMI group (**A**), RS increased in excretion frequency/week at Week 2, which was not observed in low BMI group (**B**). (**C**) The increase of excretion frequency in high and low BMI group. *p < 0.05, **p < 0.01. n = 7–12, error bars indicate SD. (**D**,**E**) In high diversity groups (**D**), RS increased in excretion frequency/week at Week 2, which was not observed in low BMI group (**E**). (**F**) The increase of excretion frequency in high and low diversity groups. *p < 0.05, **p < 0.01. n = 8–11, error bars indicate SD.

We next evaluated whether the phenotypic parameters affect RS sensitivity in humans. Participants with body mass index (BMI) higher than the total average (21.7; high BMI) exhibited a significant (*p* < 0.05) increase in the excretion frequencies after 2 weeks (5.0 ± 1.5 times/week at 0 week vs. 7.0 ± 2.0 times/week at 2 weeks in the RS group; Fig. 4A), which was not observed in lower BMI (Fig. 4B). And, at 2 weeks in the high BMI group, the excretion frequency in RS group significantly (*p* < 0.01) higher than that in placebo group (4.3 ± 1.4 times/week in placebo vs. 7.0 ± 2.0 times/week in the RS group at 2 weeks; Fig. 4A). In high BMI group, the increase in excretion frequency in RS group was significantly (*p* < 0.05) higher than that in placebo group (2.0 ± 1.8 times/week in the test group vs. − 0.1 ± 1.8 times/week in the placebo group; Fig. 4C). The excretion days per week showed the similar results in high BMI and low BMI sub-clusters (Fig. S7). Correspondingly, subjects with body weight higher than that of the total average (59.4 kg; high BW) also exhibited a significant (*p* < 0.05) increase in the excretion frequency and days (Fig. S8). Other parameters, such as age, body length, blood pressure, pulse rate, and sex did not affect RS sensitivities.

As Mancabelli et al*.* previously reported that the diversity of gut microbiota was high in constipated individuals^21^, we also categorised the participants into two groups: one with greater diversity of gut microbiota than the average diversity of all participants (121.1 species; high diversity group) and other with smaller diversity (low diversity group). RS significantly (*p* < 0.01) increased the excretion frequency per week in the high diversity group in 2 weeks (3.7 ± 1.1 times/week at week 0 vs. 5.7 ± 1.9 times/week at week 2; Fig. 4D), which was not observed in the low diversity group (Fig. 4E). RS also significantly (*p* < 0.01) increased the excretion frequency compared to the placebo group at week 2 in high diversity group (2.0 ± 1.7 times/week in RS vs. 0.0 ± 2.1 times/week in placebo at week 2; Fig. 4F). The excretion days per week showed the similar results in high diversity and low diversity sub-clusters (Fig. S9).

### Metabolic functional pathways in RS administrated subjects

**Figure 5:**
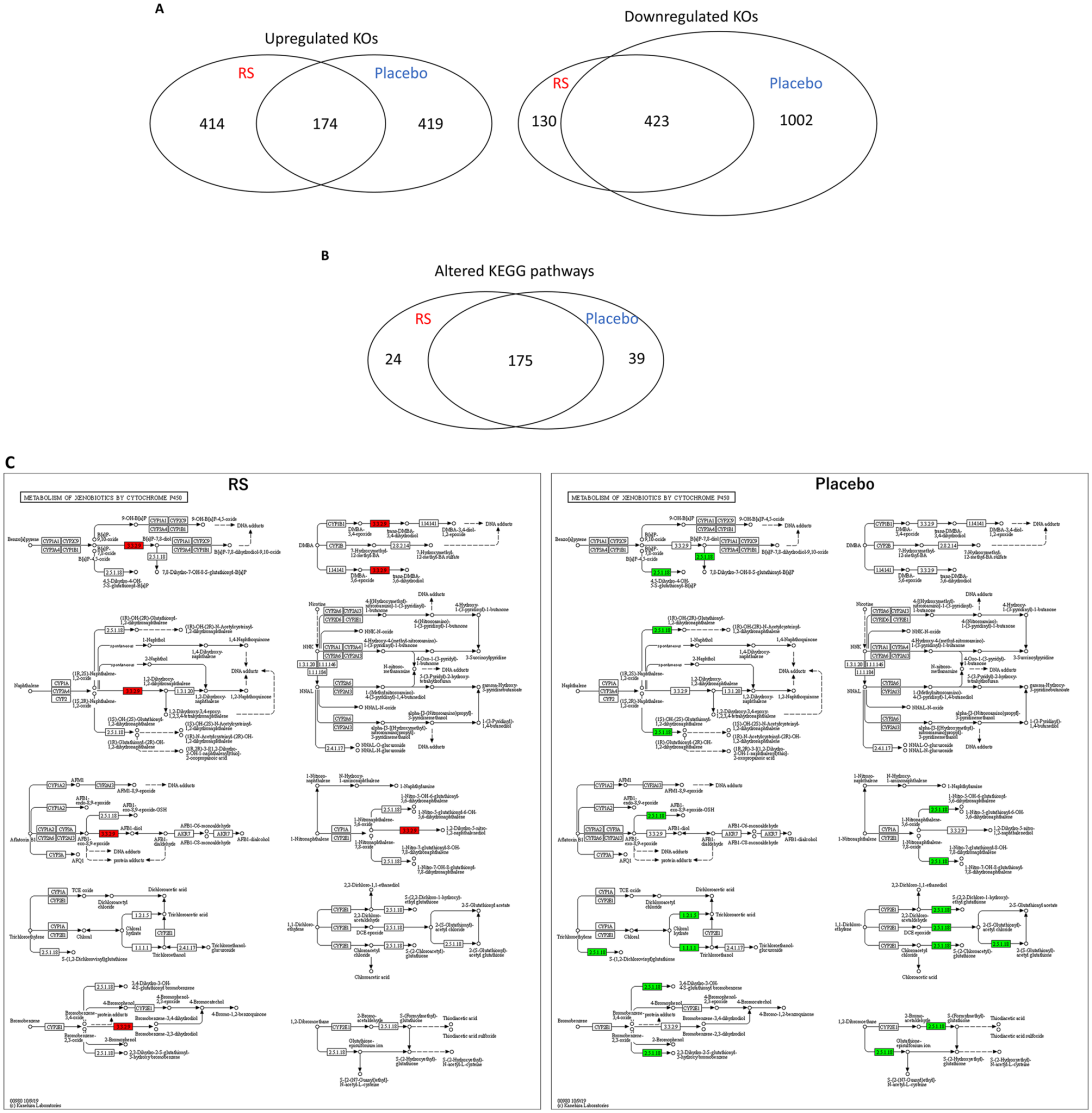

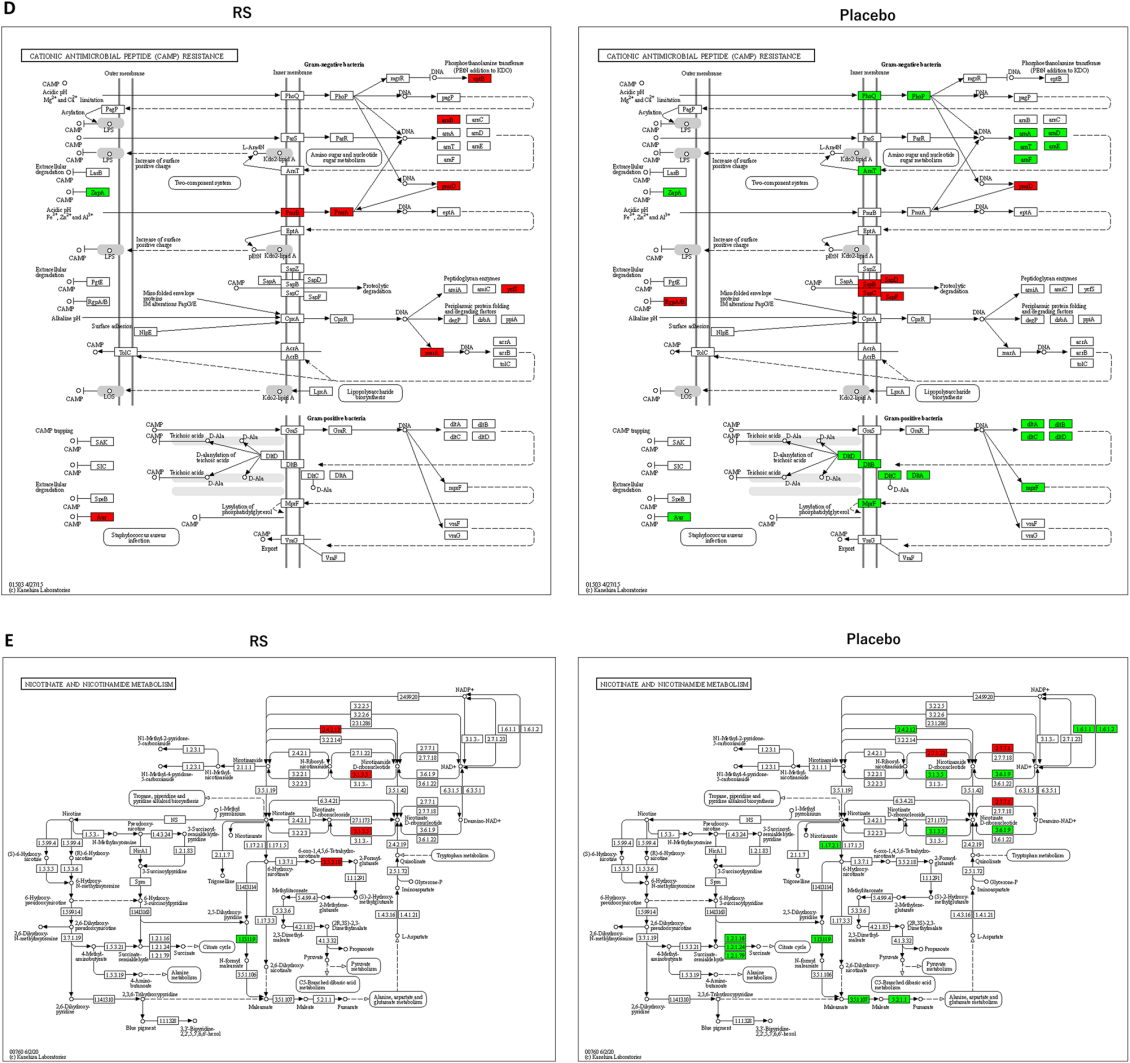
Alternation of microbial KEGG pathways in RS group. (**A**) Venn diagram analysis of altered KEGG orthologues (KOs) identified PICRUSt analysis. (**B**) Venn diagram analysis of KEGG pathways predicted by KEGG Mapper. (**C**–**E**) Several KEGG pathways were up-regulated in RS group compared with those of the placebo group. Metabolism of xenobiotics by cytochrome P450 (**C**), cationic anti-microbial peptide (CAMP) resistance (**D**), and nicotine and nicotiamide metabolism (**E**). Red and green indicates up-regulation and down-regulation, respectively.

To understand the RS-induced functional alterations in the gut microbiota of participants, bacterial metagenomes were predicted by PICRUSt using 16S rRNA sequencing, as previously reported^22^. Predicted proteins in each bacterium were classified into KEGG ortholog (KO) entities, which resulted in the identification of 6188 entities across all samples. Of these, 588 KOs and 553 KOs were up-regulated (> 2.0) and down-regulated (0.5 <) in the RS group, whereas 593 KOs and 1425 KOs were up-regulated and down-regulated in the placebo group. The up-regulated 414 KOs and the down-regulated 130 KOs were selective in RS groups (Fig. 5A). Thereafter, we mapped the differentially expressed KOs to the KEGG Mapper in order to identify the altered pathways in the participants. As illustrated in Fig. 5B, we identified 199 and 214 pathways in the RS and placebo groups, respectively. Furthermore, 175 pathways were common in both groups and 24 pathways were specific to RS administration. Because the RS-selective pathways (Table S1) contained few KOs (maximum 3 KOs in lysin biosynthesis [map00300]), we further performed different analyses. We calculated the ratio of KO counts in each KEGG pathway in the common 175 pathways and found that 56 pathways were altered in the RS group compared to placebo (> 2 or < 0.5; Table 3). After evaluating these 56 pathways manually (represented as images in supplementary materials), we selected some representative KEGG pathways altered by RS: ‘Metabolism of xenobiotics by cytochrome p450 (map00980; Fig. 5C)’, ‘Cationic anti-microbial peptide (CAMP) resistance (ko01503; Fig. 5D)’ and ‘Nicotinate and nicotinamide metabolism (map00760; Fig. 5E)’.

## Discussion

### RS improved constipation

Although we identified the therapeutic effects of RS in an HFD-induced obesity model with increased defaecation, we evaluated these effects only in constipated human subjects, and not in obese populations, as it was difficult to find obese people with constipation. Even with this limitation, we confirmed that RS increased the excretion amount both in mouse and human for the first time. In general, intake of seaweeds is beneficial for gut health and improves constipation, as they contain fibres and polysaccharides. In particular, sulphated polysaccharides from marine seaweeds affect the human microbiome^23^ and improve loperamide-induced constipation in rats^24^. Since RS is categorised in sulphated polysaccharides, our results seem reasonable for ameliorating constipation with alteration of gut microbiota.

Although RS significantly improved constipation-related phenotypes between 0 and 2-week administration (Fig. 2E,F), no significant difference in blood parameters was found between the RS and placebo groups at 2 weeks. For the Bristol scale (faeces property), the placebo group increased (*p* < 0.01) whereas RS did not, even in the high diversity group (data not shown). An increase in the Bristol scale indicates softening of faeces. Since RS retains water as a soluble fibre in faeces, this result seems inconsistent. We hypothesised that RS-induced defaecation occurred at a faster pace than water retention by RS, and that no increase was observed in the faecal water content as well as in the Bristol scale.

### RS effects on gut microbiota in various subjects

Surprisingly, the compositions of gut microbiota and KOs in the placebo group were altered more than those in the RS group (Table 2, Fig. 5). Because our trial was conducted during the first wave of the coronavirus disease 2019 (COVID-19) crisis (from February to April 2020), many participants underwent lifestyle changes such as work from home, consumed home-made meals, and avoided alcohol consumption and eating out. Unfortunately, we did not plan to record the related parameters, including the food and alcohol consumption, in addition to the mental stress. We only examined seaweed consumption (requested the participants to not eat seaweed) and safety assessment (as only one participant suffered from jammed finger) during the clinical trials. In this situation, we identified several gut bacteria that are involved in the therapeutic effect of RS. Although *Clostridiales* and *Negativicutes* were neighbouring in *the Firmicutes* phylum, *Clostridiales* decreased (Fig. 3D) and *Negativicutes* increased (Fig. 3E) in the RS group*. Clostridia* produce medium-length fatty acids that increase water absorption, and subsequently dry up faeces, causing constipation^25^. Thus, one of the therapeutic mechanisms of RS against constipation is the decrease in *the Clostridiales* class. In particular, prebiotic supplementation in constipation patients reduced the composition of *Clostridiales*^26^. RS selectively increased *Negativicutes* (Fig. 3E) and *Acidaminococcales* (Fig. 3F). Their biological effects on constipation remain unclear; however, several studies reported that the increase in these bacteria is positively related to improved constipation. *Negativicutes* were increased by Psyllium Husk (derived from seeds of *Plantago ovata*) administration^27^ and *Bifidobacterium*-based probiotic treatment^28^ during constipation improvement. *Acidaminococcus* may be one of the main factors in curing constipation during faecal microbial transplantation in clinical application^29^ and administration of partially hydrolyzed guar gum (water-soluble fibres) in children^30^. Moreover, *Veillonellales,* a pro-inflammatory and lactate-fermenting bacterium increased in the irritable bowel syndrome patients^31^, in both RS and placebo groups (Fig. 3G), thereby revealing stress from the new lifestyle due to COVID-19 crisis.

**Table 2:** Families altered during human study.

Phylum	Class	Order	Family	Time point	RS*	Placebo*	*p* value (RS vs. placebo)	*p* value (0w vs. 2w)	
								RS	Placebo
Actinobacteria	Actinobacteria	Bifidobacteriales	Bifidobacteriaceae	0W	1.75 ± 3.31	3.39 ± 5.24	0.2631	–	–
				2W	1.23 ± 1.47	1.14 ± 1.83	> 0.9999	> 0.9999	0.0798
Bacteroidetes	Bacteroidia	Bacteroidales	Bacteroidaceae	0W	30.61 ± 15.09	24.11 ± 10.40	0.273	–	–
				2W	32.14 ± 14.53	29.69 ± 12.69	> 0.9999	> 0.9999	0.4005
			Muribaculaceae	0W	0.52 ± 0.54	0.44 ± 0.41	> 0.9999	–	–
				2W	0.46 ± 0.39	0.67 ± 0.51	0.3398	> 0.9999	0.2666
Firmicutes	Clostridia	Clostridiales	Mogibacterium	0W	0.17 ± 0.24	0.30 ± 0.60	0.4883	–	–
				2W	0.09 ± 0.16	0.14 ± 0.15	> 0.9999	0.9445	0.3054
	Negativicutes	Acidaminococcales	Acidaminococcaceae	0W	1.93 ± 2.98	1.33 ± 1.82	> 0.9999	–	–
				2W	3.18 ± 3.64	1.93 ± 2.58	0.3561	0.3561	> 0.9999
		Veillonellales	Veillonellaceae	0W	1.69 ± 2.28	1.16 ± 1.34	> 0.9999	–	–
				2W	2.70 ± 2.45	2.69 ± 3.94	> 0.9999	0.4951	0.1632
Proteobacteria	Betaproteobacteria	Burkholderiales	Sutterellaceae	0W	2.53 ± 2.20	1.15 ± 1.20	0.1719	–	–
				2W	3.09 ± 3.05	2.57 ± 2.88	> 0.9999	0.9644	0.1549
Tenericutes	Mollicutes	PAC001057	PAC001057	0W	0.24 ± 0.46	0.22 ± 0.86	> 0.9999	–	–
				2W	0.05 ± 0.10	0.34 ± 1.47	0.6301	> 0.9999	> 0.9999

*The values indicate %.

### Predictive functional analysis of gut microbiota in subjects

Combination analysis using PICRUSt and KEGG Mapper was a powerful tool to predict functional alterations in gut microbiota^32^. KOs predicted by PICRUSt revealed that the number of down-regulated KOs in the placebo group was larger than that in the RS group (Fig. 5A). We mapped these KOs to the KEGG Mapper in order to predict functional pathways differentially expressed between the RS and placebo groups (Table 3, Supplementary Materials). Of these, several KOs in the pathway “metabolism of xenobiotics by cytochrome p450” were up-regulated in the RS group, whereas they were down-regulated in the placebo group (Fig. 5C). Reactions catalysed by cytochrome p450 usually turn xenobiotics, such as polysaccharides RS, to excretable metabolites^33^. This result was in accordance with that obtained in the present study. As illustrated in Fig. 5D, in “cationic anti-microbial peptide (CAMPs) resistance” pathway, several KOs were up-regulated in RS group, whereas they were down-regulated in the placebo. CAMPs are critical frontline contributors to host defence against invasive bacterial infections. For successful survival and colonisation of the host, bacteria have a series of mechanisms that interfere with CAMP activity^34^. This result suggests that RS induced CAMP resistance, which promotes the proliferation of pathogenic bacteria; however, Assoni et al. reported that CAMP resistance mediates the recovery of prominent gut commensals during inflammation^35^. In general, constipation is accompanied by inflammation in the gut mucosa, implying that RS would improve not only constipation but also mucosal damage by altering the composition of gut microbiota. Nicotinamide metabolism was down-regulated in the placebo group but up-regulated in the RS group (Fig. 5E). Nicotinamide, known as vitamin B3, is essential for life as it is part of the coenzyme NADH/NAD+, which is crucial for biological redox reactions. Moreover, it ameliorates experimental colitis in mice by improving host defence and enhancing bacterial clearance in *Citrobacter rodentium*-induced colitis^36^ and *Staphylococcus aureus* infection in mice ^37,38^. Furthermore, NAD replenishment ameliorates constipation in aged mice^39^, suggesting that one of the therapeutic mechanisms of RS is the increase in bacteria related to nicotinamide synthesis and/or secretion. In particular, our functional analysis based on PICRUSt and KEGG Mapper is just a prediction; further studies are necessary to demonstrate our speculation.

**Table 3:** Pathways differentially expressed between RS and placebo group.

KEGG	RS*	Placebo*	Ratio	Definition
map00900	1	11	0.09	Terpenoid backbone biosynthesis
map05130	1	11	0.09	Pathogenic *Escherichia coli* infection
map00550	1	10	0.10	Peptidoglycan biosynthesis
map00984	1	9	0.11	Steroid degradation
map03070	4	35	0.11	Bacterial secretion system
map00480	1	8	0.13	Glutathione metabolism
map00020	1	7	0.14	Citrate cycle (TCA cycle)
map00906	1	6	0.17	Carotenoid biosynthesis
map05150	1	6	0.17	*Staphylococcus aureus* infection
map00130	3	16	0.19	Ubiquinone and other terpenoid-quinone biosynthesis
map00982	1	5	0.20	Drug metabolism—cytochrome P450
map00010	3	14	0.21	Glycolysis/gluconeogenesis
map00030	3	14	0.21	Pentose phosphate pathway
map00310	2	9	0.22	Lysine degradation
map00220	2	8	0.25	Arginine biosynthesis
map00250	2	8	0.25	Alanine, aspartate and glutamate metabolism
map00710	1	4	0.25	Carbon fixation in photosynthetic organisms
map00980	1	4	0.25	Metabolism of xenobiotics by cytochrome P450
map05132	1	4	0.25	Salmonella infection
map05133	1	4	0.25	Pertussis
map00620	5	18	0.28	Pyruvate metabolism
map02025	7	25	0.28	Biofilm formation—*Pseudomonas aeruginosa*
map00561	2	7	0.29	Glycerolipid metabolism
map05131	2	7	0.29	Shigellosis
map00910	5	16	0.31	Nitrogen metabolism
map00311	1	3	0.33	Penicillin and cephalosporin biosynthesis
map00460	1	3	0.33	Cyanoamino acid metabolism
map00472	1	3	0.33	d-Arginine and d-ornithine metabolism
map00626	1	3	0.33	Naphthalene degradation
map00770	1	3	0.33	Pantothenate and CoA biosynthesis
map00903	1	3	0.33	Limonene and pinene degradation
map02030	2	6	0.33	Bacterial chemotaxis
map03410	1	3	0.33	Base excision repair
map05134	1	3	0.33	Legionellosis
map05204	1	3	0.33	Chemical carcinogenesis
map05225	1	3	0.33	Hepatocellular carcinoma
map05418	1	3	0.33	Fluid shear stress and atherosclerosis
map00640	8	23	0.35	Propanoate metabolism
map01501	7	19	0.37	beta-Lactam resistance
map00364	3	8	0.38	Fluorobenzoate degradation
map05100	3	8	0.38	Bacterial invasion of epithelial cells
map00760	5	13	0.38	Nicotinate and nicotinamide metabolism
map05111	6	15	0.40	Biofilm formation—*Vibrio cholerae*
map00051	9	22	0.41	Fructose and mannose metabolism
map01200	15	36	0.42	Carbon metabolism
map00340	3	7	0.43	Histidine metabolism
map00540	6	14	0.43	Lipopolysaccharide biosynthesis
map01503	9	20	0.45	Cationic antimicrobial peptide (CAMP) resistance
map01110	66	146	0.45	Biosynthesis of secondary metabolites
map00650	10	22	0.45	Butanoate metabolism
map00790	5	11	0.45	Folate biosynthesis
map01212	5	11	0.45	Fatty acid metabolism
map00350	9	19	0.47	Tyrosine metabolism
map02026	10	21	0.48	Biofilm formation—*Escherichia coli*
map02010	53	111	0.48	ABC transporters
map01100	282	567	0.50	Metabolic pathways

*The values indicate the numbers of KOs in each pathway.

### Limitation

This study has several limitations. First, the duration of RS administration was limited to only 2 weeks, which seems insufficient to evaluate the long-term efficacy. Second, this trial was performed on the non-obese adults. Our analysis indicates that the body weight is positively correlated with RS efficacy, even in the range comprising healthy individuals. For example, we evaluated the presence of fucoidan, a sulphated polysaccharide extracted from seaweeds in overweight or obese adults for 3 months, and demonstrated its efficacy in disease phenotypes^40^, and it was expected to improve gut dysbiosis^41^. Third, this was a single-centre focused study, and the sample size was relatively small.

## Conclusion

RS promoted defaecation in mice and human subjects without any side effects and improved gut microbiota. Therefore, co-administration of RS with other prebiotics and probiotics may enhance this effect in future studies. In conclusion, with other health-promoting properties of RS (lipid-lowering, anti-viral and anti-thrombotic), it is a powerful constituent in *M. nitidum,* and can be used as a therapeutic or preventive supplement due to its anti-constipation properties.

## Methods

### Preparation of rhamnan sulphate (RS)

For mouse experiments, we used purified RS (> 95% purity) as previously described^13^. For the clinical study, Rhamnox (Konan Chemical Manufacturing, Mie, Japan) was used for RS. The preparation of Rhamnox is described as follows according to a previous study^15^, with certain modifications. Dried *M. nitidum* (700 g) was washed with H_2_O, by adding up to 18 L of H_2_O, and then extracted for 6 h at 100 °C. To prevent foaming, 2–5 g of citrate acid was added after 40 min of heating. Thereafter, celite (540 g; Radiolite 900; Showa Chemical Industry, Tokyo, Japan) was added to the extract, and then centrifuged to remove the algal fronds. Next, celite (90 g) was re-added to the extract and filtered with qualitative filter paper grade 2 (Pellicon Cassette System P2B010V01; Millipore, Billerica, MA, USA). Hot water extract obtained in this manner was fractionated via ultrafiltration (Millipore; cutoff MW 10,000). The extract was sterilised at 85–90 °C for 15 min and then dried using a spray drier (Ohkawara Kakohki, Kanagawa, Japan) to yield ~ 200 g of the extract. The RS content determined by gel permeation chromatography using Shodex OHpac SB-G 6B and SB-803HQ columns (eluent: 0.1 M KCl, 0.15 M KH_2_PO_4_ K_2_HPO_4_ buffer pH6.7) was 87.3% compared to the reference standard, which is the purified RS, as previously described^13^. Additionally, 6.3% impurity was identified as water.

### Ethics

All animal procedures were approved by the Ethics Committee of Mie University and were performed according to the Japanese Animal Welfare Regulation ‘Act on Welfare and Management of Animals’ (Ministry of Environment of Japan) and complied with international guidelines. The clinical study was conducted according to the guidelines laid down in the Declaration of Helsinki, and all procedures involving human subjects were approved by the Institutional Review Board of Chiyoda Paramedical Care Clinic (Tokyo, Japan). Written informed consent was obtained from all subjects. This study was carried out in compliance with the ARRIVE guidelines (https://arriveguidelines.org/) and was registered in the University Hospital Medical Information Network Clinical Trials Registry (UMINCTR: https://upload.umin.ac.jp/cgi-open-bin/ctr_e/ctr_view.cgi?recptno=R000045150) as Number UMIN000039591 (date of registration: 25/2/2020).

### Mouse experiment

NSY/HOS mice, a type 2 diabetes mellitus strain^42^, was purchased from Hoshino Laboratory Animals (Saitama, Japan) and housed on a 12-h light/dark cycle at the Institute of Laboratory Animals at Mie University. Six-month-old male mice were assigned to three groups with six mice, housed individually, and were fed either the CE-7 normal diet (ND; CLEA Japan, Tokyo, Japan), high-fat diet (Test Diet 58Y1; TestDiet, Richmond, IN, USA) or a high-fat diet (HFD) supplemented with RS (250 mg/Kg BW) for 4 weeks to induce obesity. The compositions of ND and HFD are described in Table S2. During the feeding experiment, body weight, food intake, and faecal weight were measured once per week. Mice were subjected to fasting for 14 h before blood sampling to assess the fasting blood glucose levels. Blood glucose was measured using a hand-held glucometer (Glutest Neo Super; Sanwa Kagaku, Nagoya, Japan). The plasma levels of triacylglycerol (TG), low-density lipoprotein cholesterol (LDL-C), and total cholesterol (TCHO) were measured using Wako L-type TG, Wako L-type LDL-C, and Wako L-type TCHO (Wako Pure Chemicals, Osaka, Japan) assay kits according to the manufacturer’s protocol. The mice were euthanized with CO_2_ gas, and then the organ samples were collected and subsequently dissected for analysis.

### Caloric analysis of mouse stools

The stool samples were collected daily and stored at − 20 °C. The nutritional composition (fat, protein, moisture, ash, carbohydrate and energy) was determined as described elsewhere^43^ with certain modifications. The fat, protein, moisture, and ash contents were evaluated by the Folch method, Kjeldahl method, atmospheric heating drying method and by direct ashing method, respectively; moreover, the carbohydrate content was assessed by subtracting the fat, protein, moisture and ash contents from the total amount. Energy content was calculated using modified Atwater factors (4, 9 and 4 kcal/g for protein, fat, and carbohydrate, respectively).

### Design of clinical study

This was a randomised, double-blind, placebo-controlled, and parallel-group study carried out in a single clinical centre (Chiyoda Paramedical Care Clinic; CPCC, Tokyo, Japan) in Japan. Mie University Graduate School of Medicine and Konan Chemical Manufacturing together prepared the study protocol. All study procedures were undertaken by a clinical research organisation (CPCC) on consignment from Konan Chemical.

### Subjects

Seventy-three healthy Japanese male and female volunteers (20–65 years) were selected from the total volunteers registered in the CPCC. The study details were disclosed to the subjects before enrolment, and the investigators obtained their written informed consent. Thereafter, the subjects underwent various tests (lifestyle questionnaire, medical interview, physiological, biochemical, and haematological tests). Each of these tests was performed at the CPCC. Subjects who did not meet the exclusion criteria, but met the inclusion criteria, were enrolled in the 2-week screening. Selected subjects recorded their defaecation status (number of defaecations, date of defaecation, confirmation of how many defaecations were collected on the day of defaecation, Bristol scale (faecal properties), amount of defaecation, colour of stool, smell of stool, refreshing feeling at the time of defaecation and stomach condition (abdominal pain, swelling, gurgling, bloating, flatulence, nausea) in a diary. Health adults aged 20–65 years who had relatively low defaecation frequencies (3–5 times/week) were selected for this study. There were 15 exclusion criteria, as described in Table S3. Eventually, 38 subjects were selected as the study subjects.

### Randomization

Subjects were randomly allocated into the RS or placebo group to balance the sex, age, faecal frequency and BSS. Allocation was operated by a researcher of the CPCC who was not involved in taking measurements and analysis.

### Blinding

In total, 100 mg of RS (Rhamnox) was filled in a cellulose white capsule (Matsuya, Osaka, Japan). The placebo involved an empty capsule, identical in appearance and flavour, and was then provided to CPCC with a coded name. Correspondence of the coded name and the true name of the product was disclosed to CPCC after completion of data analysis.

### Study protocol

After a 3-week screening, 19 and 19 participants were allocated to the RS or placebo group, respectively. Each participant ingested 1 capsule/day for 2 weeks. After the end of the trial, no participant experienced adverse events. Participants recorded their life survey and defaecation questionnaire in diary every day during the study period. The test schedule is illustrated in Fig. S1.

### Data collection

The primary outcomes were defaecation frequency, defaecation date and Bristol Scale. The secondary outcomes were gut microbiota and faecal condition (faecal odour, colour, volume and feeling after defaecation). Faecal frequency and condition were assessed by recording the defaecation times and faecal condition every day in a diary. Analysis exclusion criteria are described in Table S4; however, no subject was excluded from the analysis.

### Faecal sample collection and DNA extraction

Faecal samples were collected using a guanidine thiocyanate solution (Faeces Collection kit; Techno Suruga Lab, Shizuoka, Japan). After vigorous mixing, the samples were stored at 4 °C for a maximum of 7 days until DNA extraction. After homogenisation with lysis solution F (Nippon Gene, Tokyo, Japan), the genomic DNA was heated at 65 °C for 10 min and purified from the supernatants using the MPure Bacterial DNA Extraction Kit (MP Biomedicals, Solon, OH, USA) with MPure-12 system (MP Biomedicals). The purified DNA was quantified using Synergy LX (BioTek Instruments, Winooski, VT, USA) and QuantiFluor system (Promega, Madison, WI, USA).

### Sequencing of 16S rRNA gene

Illumina MiSeq paired-end sequencing of the hypervariable V3–V4 regions of the 16S rRNA was performed at Bioengineering Lab. Co., Ltd. (Kanagawa, Japan). A two-step, tailed PCR approach was used according to the protocol for 16S metagenomic sequencing library preparation (Illumina, San Diego, CA, USA). Both the V3 and V4 regions of the 16S ribosomal RNA were amplified with primers containing the Illumina overhang adaptor (forward primer 5′ ACA CTC TTT CCC TAC ACG ACG CTC TTC CGA TCT CCT ACG GGN GGC WGC AG; Reverse primer 5′ GTG ACT GGA GTT CAG ACG TGT GCT CTT CCG ATC TGA CTA CHV GGG TAT CTA ATC C). Thereafter, Index PCR was performed with Index 1 and Index 2 primers from the Nextera XT Index Kit (Illumina), using 2 μL of amplicon derived from the previous PCR. Next, the indexed libraries were cleaned and analysed with a Fragment Analyser, using a dsDNA 915 Reagent Kit (Advanced Analytical Technologies, Ames, IA, USA). The prepared libraries were used for paired-end sequencing using MiSeq v3 reagents and 2 × 300-bp reads on the MiSeq (Illumina).

### Analysis of bacterial composition in 16S rRNA datasets

The paired-end reads of the 16S rRNA gene were assembled using QIIME2 (ver. 2020.2), with the default parameter values, were applied for sequence de-noising, primer sequence trimming and chimaera checking using the DADA2 method^44,45^. Quality filtered reads were assigned to operational taxonomic units (OTUs) (100% identity) using de novo OTU picking and taxonomic assignment using the feature-classifier plug-inn against the EzBioCloud 16S database (https://www.ezbiocloud.net/)^46^.

### Microbiome analysis

Diversity analyses were performed using QIIME with default parameters. α-diversity using Chao1 index was analysed based on the size of samples and normalised using the minimum number of sequences obtained among different samples. β-diversity across the samples was calculated based on the unweighted UniFrac^47^ distance matrices using the script beta_diversity_through_plots.py. A principal coordinate analysis (PCoA) plot was constructed to present the overall dissimilarity in the bacterial communities of different groups.

Phylogenetic Investigation of Communities by Reconstruction of Unobserved States (PICRUSt2, version 2.3.0b)^48^ was used to predict the functional gene products (protein) in the faecal microbiota based on the taxonomy obtained from the EzBioCloud 16S database. The output contains gene products with their respective counts in each bacterium of the participant. The gene products were classified using KEGG ortholog (KO), and categorised as up- (> 2.0) and down-regulated (< 0.5) KOs based on their corresponding values in the RS group compared to the placebo group. Subsequently, the acquired KEGG orthologs were mapped using the KEGG Mapper^49^ as previously reported^32^.

### Safety assessment

The principal investigator assessed the safety of RS based on the results of participant communication, tests (physiological, biochemical, haematological) and urinalyses. The daily diary content was also referred to for safety assessment.

### Sample size

Although RS was tested on subjects with constipation, no testing was performed on these healthy subjects; hence, we were unable to estimate the minimum number of subjects. Thereafter, we set the minimum number of subjects to 19 for statistical analysis.

### Statistical analysis

All results were represented as means with standard deviations (SD). Data except increase of excretion frequency and days (Fig. 2F, Supplemental Fig. S3) were analysed using the two-way repeated-measures analysis of variance (ANOVA) with the Bonferroni *post-hoc* test to to compare the different groups, using GraphPad Prism version 7 (GraphPad Software, San Diego, CA, USA) or IBM SPSS software (IBM, Armonk, NY, USA). The increase of excretion frequency and days were analysed using Student’s t-test, using GraphPad Prism version 7. For sub-cluster analysis, we categorised the participants into two groups: one with values greater than the average values of all participants and other with smaller values for each parameter.

## Supplementary Information

Supplementary Figures.

Supplementary Movie.

Supplementary Tables.
