# Supplementary material for: Rhamnan sulphate from green algae Monostroma nitidum improves constipation with gut microbiome alteration in double-blind placebo-controlled trial
Source: Sci Rep. 2021 Jul 5;11:13384. doi: 10.1038/s41598-021-92459-7 (PMC8257721; doi:10.1038/s41598-021-92459-7)
Supplement: Supplementary file 1 — Supplementary Figures. [file 41598_2021_92459_MOESM1_ESM.docx]

**Table S1. Pathways derived from RS-selective KOs.**

| KEGG pathway | Definition | #KO* |
| --- | --- | --- |
| map00300 | Lysine biosynthesis | 3 |
| map00523 | Polyketide sugar unit biosynthesis | 2 |
| map03060 | Protein export | 2 |
| map00121 | Secondary bile acid biosynthesis | 1 |
| map00524 | Neomycin, kanamycin and gentamicin biosynthesis | 1 |
| map00565 | Ether lipid metabolism | 1 |
| map00940 | Phenylpropanoid biosynthesis | 1 |
| map00941 | Flavonoid biosynthesis | 1 |
| map00945 | Stilbenoid, diarylheptanoid and gingerol biosynthesis | 1 |
| map01055 | Biosynthesis of vancomycin group antibiotics | 1 |
| map03010 | Ribosome | 1 |
| map03013 | RNA transport | 1 |
| map03015 | mRNA surveillance pathway | 1 |
| map03040 | Spliceosome | 1 |
| map04070 | Phosphatidylinositol signaling system | 1 |
| map04080 | Neuroactive ligand-receptor interaction | 1 |
| map04120 | Ubiquitin mediated proteolysis | 1 |
| map04137 | Mitophagy–animal | 1 |
| map04657 | IL-17 signaling pathway | 1 |
| map04723 | Retrograde endocannabinoid signaling | 1 |
| map04725 | Cholinergic synapse | 1 |
| map04970 | Salivary secretion | 1 |
| map04976 | Bile secretion | 1 |
| map05171 | Coronavirus disease—COVID-19 | 1 |

**Table S2. Nutritional components of mouse feed.**

|  | Control diet (CE-7) | HFD (58Y1) |
| --- | --- | --- |
| Protein | 17.9 | 23.1 |
| Fat | 3.7 | 34.9 |
| Fibre | 5.1 | 6.5 |
| Carbohydrates | 58.9^*^ | 25.9 |
| Ash | 6.4 | 3.8 |
| Water | 8.1 | 5.7^*^ |
|  |  |  |
| Energy (kcal/g) | 3.4 | 5.1 |

* Calculated from other components.

**Table S3. Exclusion criteria.**

| Regularly eat seaweeds, *Monostroma nitidum* |
| --- |
| Undergoing drug treatment |
| Taking foods with functional claims, and health foods at least thrice a week |
| Pregnant or planning to become pregnant or are breastfeeding |
| A history of serious diseases such as heart, liver, kidneys, digestive system, etc. |
| Alcohol polydipsia |
| Extremely irregular eating habits and lifestyles |
| Allergic to medicines and food |
| Participating in clinical trials of other medicines or health foods; planning to participate in other clinical trials within 4 weeks after the study |
| Donated component blood or 200 mL of whole blood a month before the study |
| Donated 400 mL of whole blood 3 months (male) or 4 months (female) before the study |
| Donated a total blood sampling volume of more than 1200 mL (male) or 800 mL (female) 12 months before the study |
| Denied to participate in this study by the principal investigator or the investigator. |

**Table S4. Analysis exclusion criteria.**

| Consumed less than 85% of the intake rate of RS or placebo |
| --- |
| Filled in diary less than 85% |
| Did not follow the required restrictions |
| Presented various side effects that impair the reliability of the clinical data |
| Met exclusion criteria after the study |
| Presented clear reason for exclusion |
