# Supplementary material for: Rhamnan sulphate from green algae Monostroma nitidum improves constipation with gut microbiome alteration in double-blind placebo-controlled trial
Source: Sci Rep. 2021 Jul 5;11:13384. doi: 10.1038/s41598-021-92459-7 (PMC8257721; doi:10.1038/s41598-021-92459-7)
Supplement: Supplementary file 3 — Supplementary Tables. [file 41598_2021_92459_MOESM3_ESM.docx]

**Figure S1**


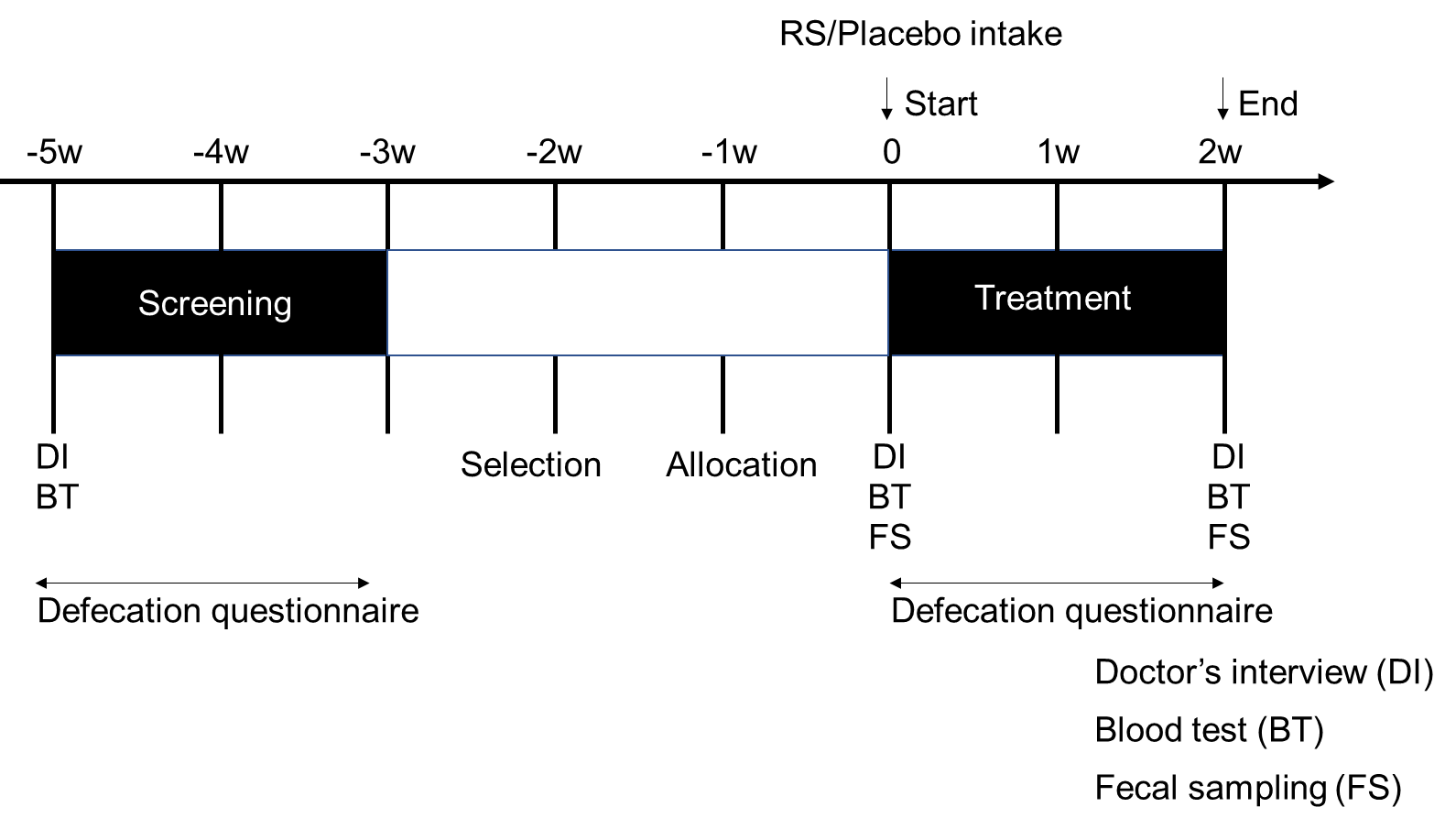


**Figure S1. Test schedule for human trial.**

**Figure S2**

**Figure S2. Flow chart of participant selection.**

**Figure S3**


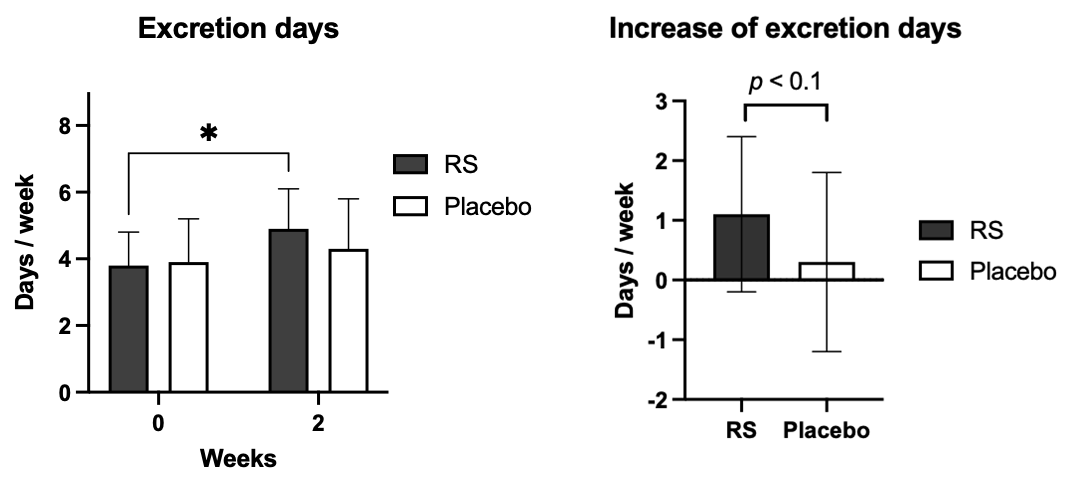


**Figure S3. RS increases excretion days per week.** **p* < 0.05. *n* = 19, error bars indicate SD.

**Figure S4**


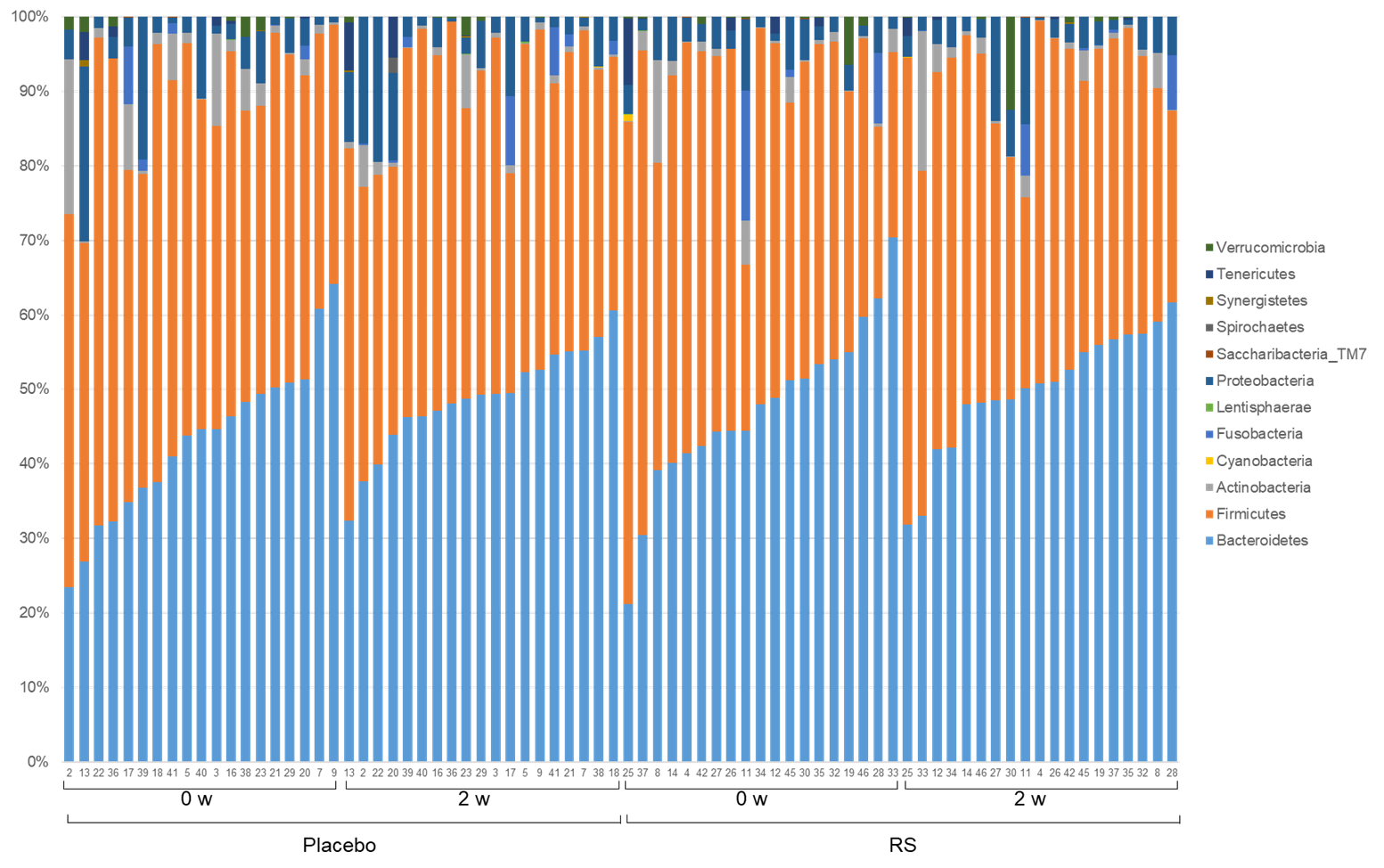


**Figure S4. Taxonomy summary (phylum level)**

**Figure S5**

**
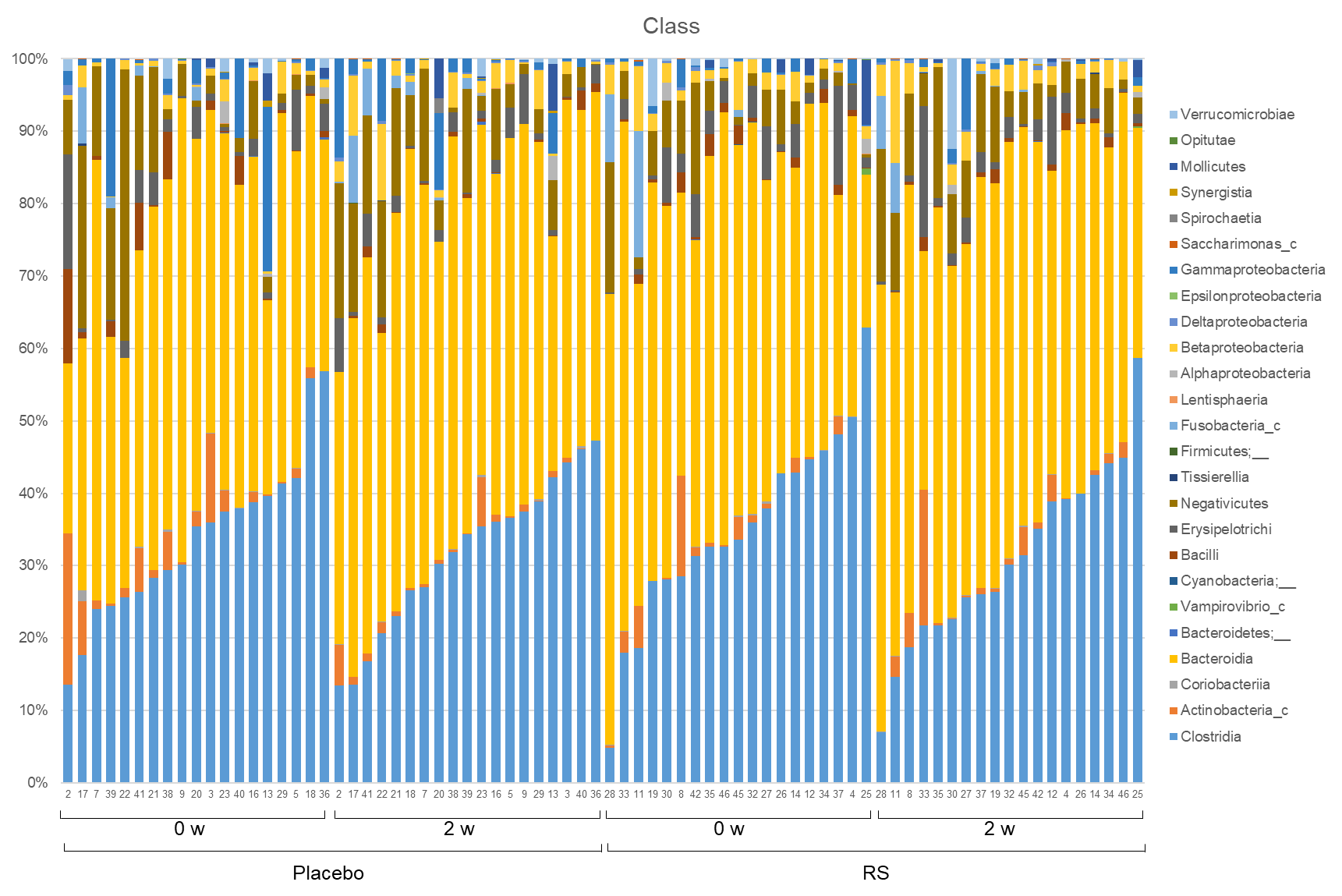
**

**Figure S5. Taxonomy summary (class level)**

**Figure S6**

**
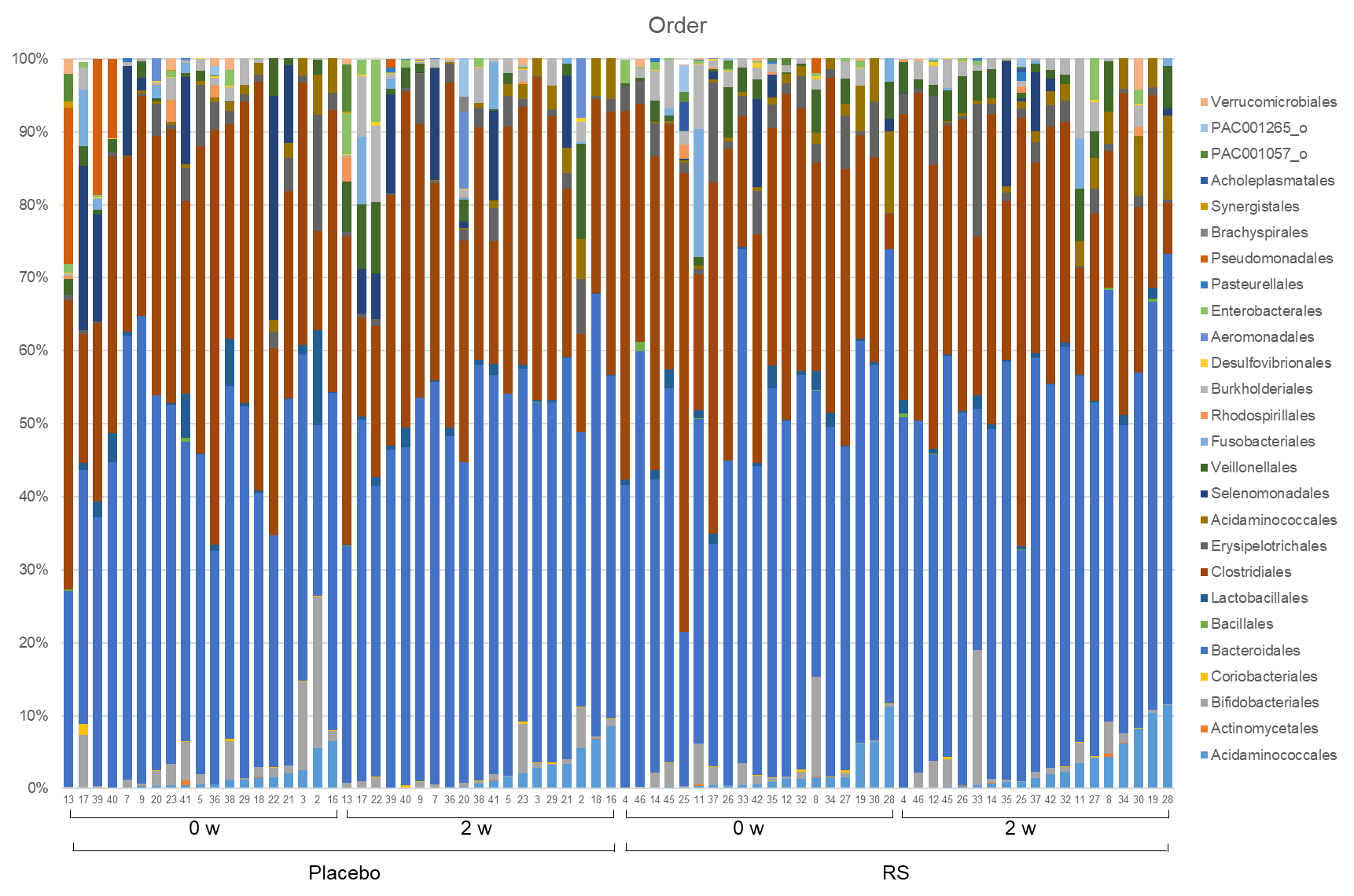
**

**Figure S6. Taxonomy summary (order level)**

**Figure S7**

**
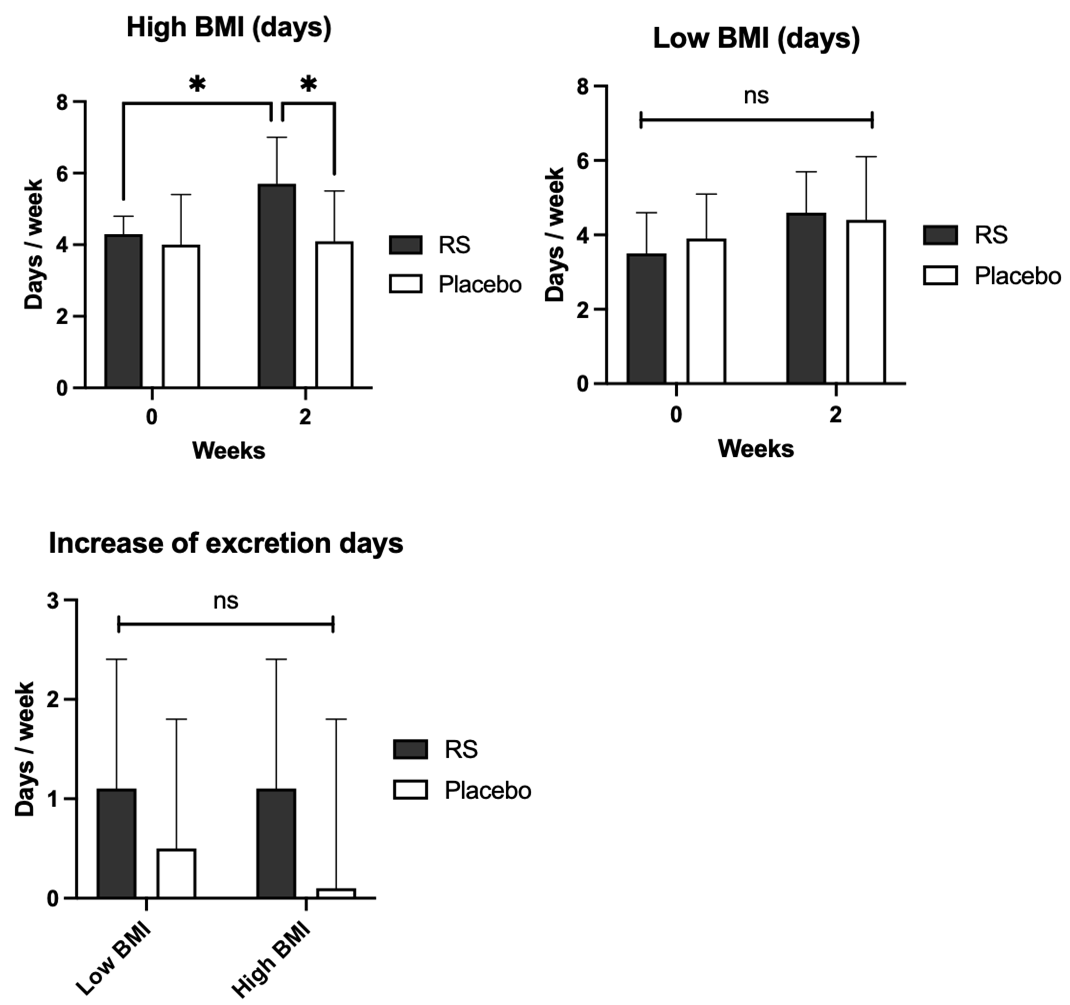
**

**Figure 7. Sub-cluster analysis of excretion days based on BMI.** **p* < 0.05. *n* = 7-12, error bars indicate SD.

**Figure S8**

**
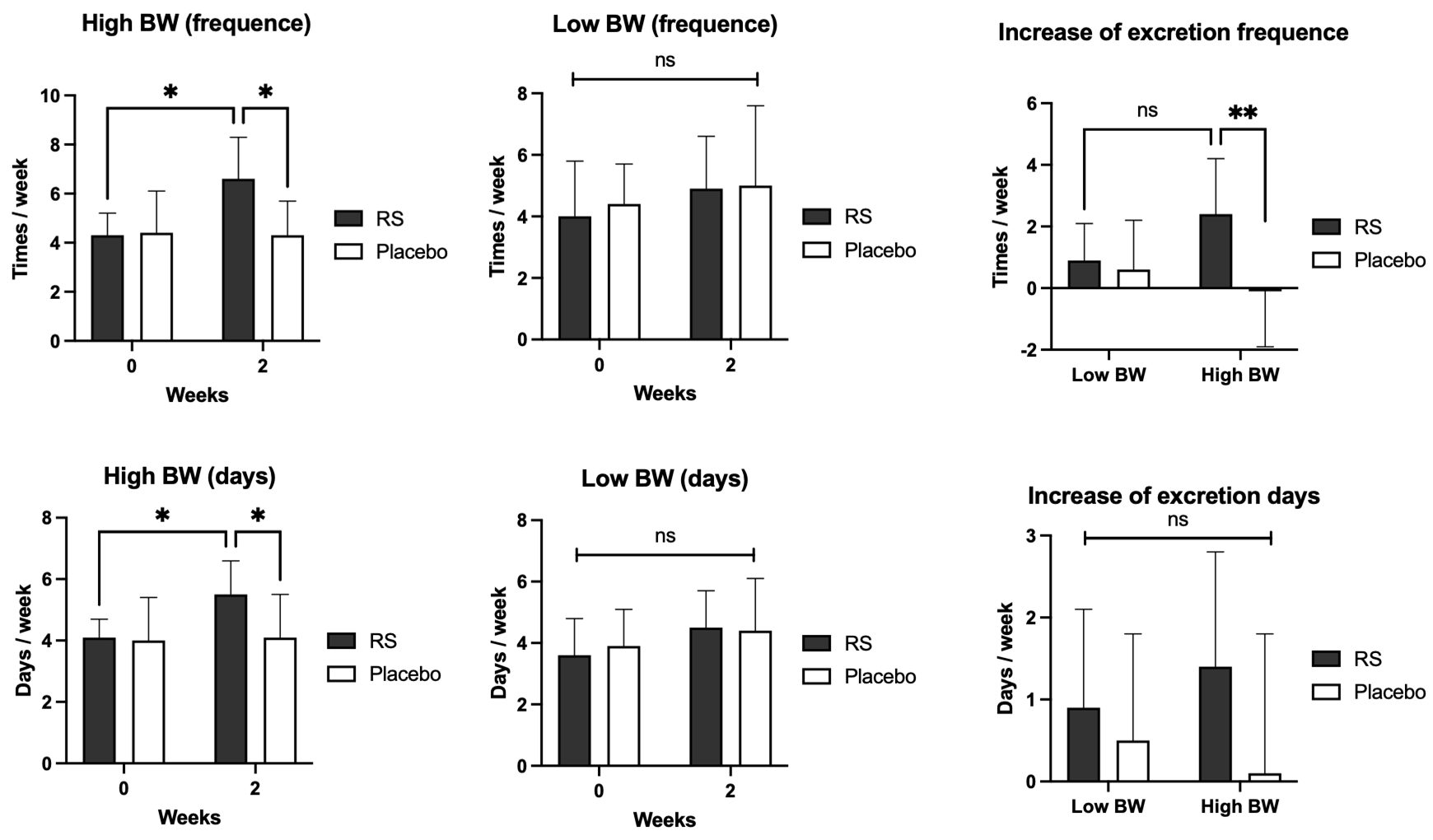
**

**Figure S8. Sub-cluster analysis of constipated phenotypes based on body weight (BW).** **p* < 0.05, ***p* < 0.01. *n* = 8-11, error bars indicate SD.

**Figure 9**

**
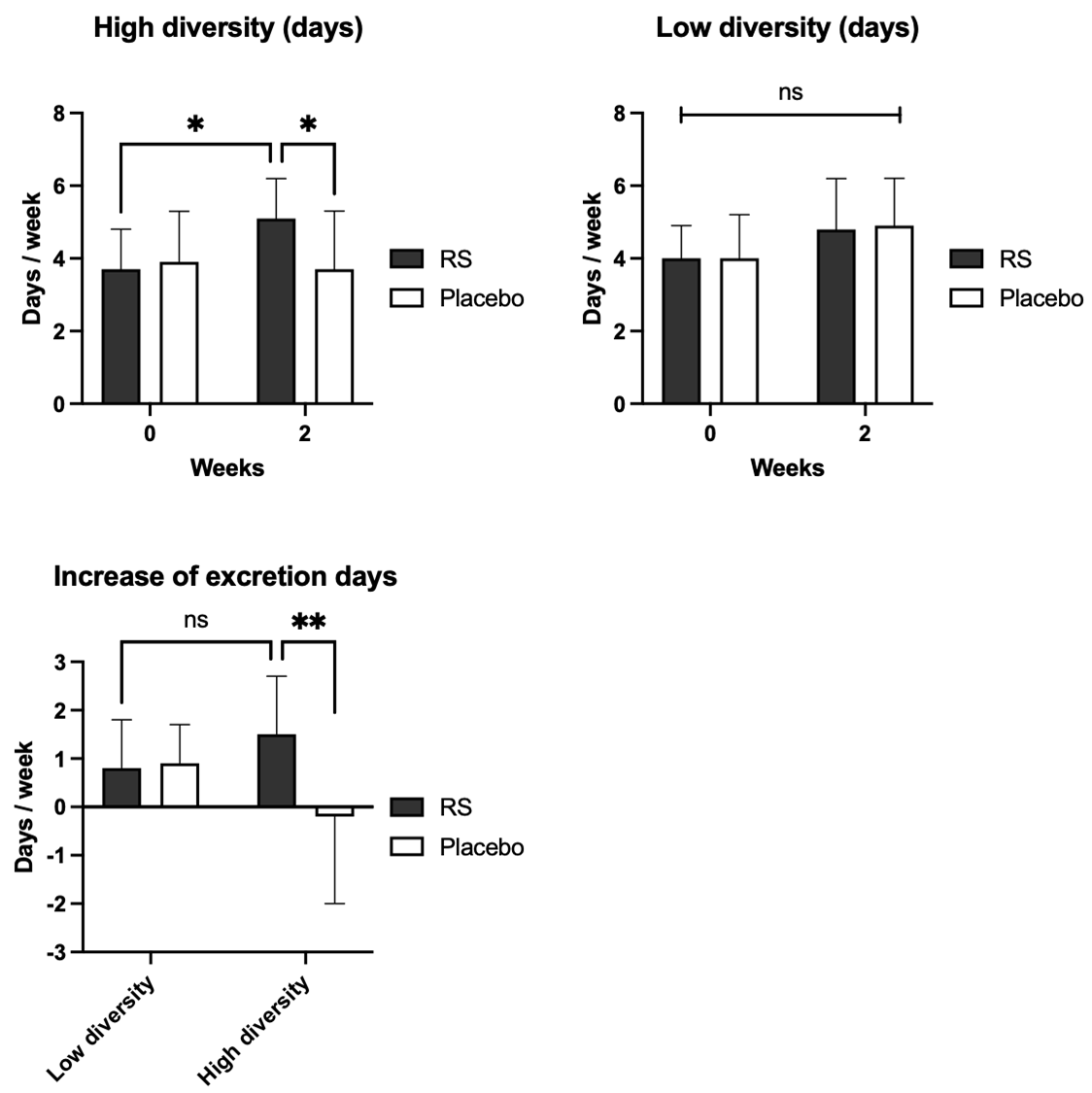
**

**Figure S9.** **Sub-cluster analysis of excretion days based on bacterial diversity.** **p* < 0.05, ***p* < 0.01. *n* = 8-11, error bars indicate SD.
